# Mobile phone use, exposure to radiofrequency electromagnetic field, and brain tumour: a case–control study

**DOI:** 10.1038/sj.bjc.6604214

**Published:** 2008-02-05

**Authors:** T Takebayashi, N Varsier, Y Kikuchi, K Wake, M Taki, S Watanabe, S Akiba, N Yamaguchi

**Affiliations:** 1 Department of Preventive Medicine and Public Health, Keio University School of Medicine, Tokyo, Japan; 2 Department of Electrical and Electronic Engineering, Tokyo Metropolitan University, Tokyo, Japan; 3 EMC Group, Applied Electromagnetic Engineering, National Institute of Information and Communications Technology, Tokyo, Japan; 4 Department of Epidemiology and Preventive Medicine, Kagoshima University Graduate School of Medical and Dental Sciences, Kagoshima, Kagoshima; 5 Department of Public Health, Tokyo Women's Medical University, Tokyo 162-8666, Japan

**Keywords:** glioma, meningioma, mobile phone, case–control study, epidemiology

## Abstract

In a case–control study in Japan of brain tumours in relation to mobile phone use, we used a novel approach for estimating the specific absorption rate (SAR) inside the tumour, taking account of spatial relationships between tumour localisation and intracranial radiofrequency distribution. Personal interviews were carried out with 88 patients with glioma, 132 with meningioma, and 102 with pituitary adenoma (322 cases in total), and with 683 individually matched controls. All maximal SAR values were below 0.1 W kg^−1^, far lower than the level at which thermal effects may occur, the adjusted odds ratios (ORs) for regular mobile phone users being 1.22 (95% confidence interval (CI): 0.63–2.37) for glioma and 0.70 (0.42–1.16) for meningioma. When the maximal SAR value inside the tumour tissue was accounted for in the exposure indices, the overall OR was again not increased and there was no significant trend towards an increasing OR in relation to SAR-derived exposure indices. A non-significant increase in OR among glioma patients in the heavily exposed group may reflect recall bias.

The rapid increase in mobile phone use has raised public concern about their safety (Rothman *et al*, 1996; Violanti and Marshall, 1996; Repacholi, 1998; Blettner and Berg, 2000; Rothman, 2000). Since only glial and meningial tissue close to the surface of the head is exposed to relatively high electromagnetic fields (EMFs) emitted from mobile phones, brain tumours, especially glioma and meningioma, have received particular attention, along with acoustic neurinoma and salivary gland tumours. To investigate whether mobile phone users have an increased risk for these tumours, a collaborative case–control study in 13 countries, the INTERPHONE study, was initiated in 2000, coordinated by the International Agency for Research on Cancer (IARC) and is still underway (Cardis and Kilkenny, 1999; Christensen *et al*, 2005; Lönn *et al*, 2005; Hepworth *et al*, 2006; Schuz *et al*, 2006; Takebayashi *et al*, 2006; Cardis *et al*, 2007; Hours *et al*, 2007). Some national reports are already published, with mixed findings.

A central issue has been how precisely to estimate the actual EMF exposure, given the necessary reliance on self-reported use. Different parts of the brain are known to be exposed to EMFs of different magnitudes, related not only to which ear the phone is placed on, but also to the characteristics of different mobile phone models. The specific absorption rate (SAR) is widely accepted as a dosimetric quantity in guidelines on EMF exposure (e.g., the International Commission on Non-Ionizing Radiation Protection (ICNIRP), 1998) in frequency ranges including those used for mobile phones. The SAR, representing absorbed radiofrequency (RF) power per unit mass of body tissue, is closely related to thermal effects. If non-thermal effects are involved, SAR is also relevant, being as it is closely correlated with internal electric and magnetic fields in tissue near the radiation source (Mochizuki *et al*, 2002).

We have investigated whether mobile phone use increased brain tumour risk in Japan. The study followed the common, core protocol of the INTERPHONE study, but for estimating RF exposure level in different areas of the brain in light of its great variability, we adopted a new approach using a heterogeneous head model (Japanese numerical TARO model’s head) (Nagaoka *et al*, 2004).

## METHODS

The study area encompassed Tokyo, consisting of 23 wards (the metropolitan area) and 14 cities (the municipal area), along with 25 adjacent cities. In a preliminary survey, it was found that 30 of the 172 hospital neurosurgery departments in Tokyo treated approximately 90% of brain tumours in the area. Of these 30 departments, 21 agreed to participate in the study, and so it was estimated that about 75% of the meningioma and glioma cases in the study area would be covered. There were no differences in patients’ socioeconomic status between participating and non-participating hospitals. The case group consisted of newly diagnosed meningiomas, gliomas, and pituitary adenomas at ages 30–69 years treated in participating hospitals, with case recruitment performed prospectively from December 2000 to November 2004. Participation was 58.7% (88 out of 150) for glioma, 77.6% (132 out of 170) for meningioma, and 75.6% (102 out of 135) for pituitary adenoma. The main reason for non-participation was failure to contact the patients; for example, only 5% of glioma eligible patients refused, whereas the rest (37%) were not informed of the study during their hospital stay by their attending physicians. The 10 cases found to have been diagnosed before June 2000, more than 6 months before the study began, were excluded from the analysis (with their controls) leaving 83 gliomas, 128 meningiomas, and 101 pituitary adenomas. Cases treated surgically were verified histopathologically (codes of ICD-O 3rd ed.: glioma, 9380–9384, 9390–9394, 9400–9401, 9410–9411, 9420–9424, 9430, 9440–9443, 9450–9451, 9460, 9480–9481; meningioma, 9530–9539), but some were diagnosed by magnetic resonance imaging. Among the 83 gliomas and 128 meningiomas, 78 (94%) and 118 (92.2%), respectively, underwent surgery. The date of diagnosis was defined as when the tumour was first identified radiologically.

Controls were selected from the general population by random digit dialling, in which phone numbers for home fixed-phones were generated randomly. Volunteers to act as controls were sought until at least one for each case was identified who agreed to participate; they were individually matched for age (within a 5-year range), sex, and residence with the cases. Participation among contacted controls was 52.5% (196 out of 373) for glioma, 51.6% (279 out of 541) for meningioma, and 49.4% (208 out of 421) for pituitary adenoma. A brief questionnaire was administered over the phone or via a self-administered paper questionnaire to those who did not agree to face-to-face interviews, to compare phone use between participants and non-participants among eligible controls. Basic information on age, sex, brief history of mobile phone use, and some life-style factors was available for 75.6, 73.0, and 72.0% of the control for glioma, meningioma, and pituitary cases, respectively.

Each case subject and matched control was interviewed by the same nurse or other health professional specifically trained for this study. A Japanese version of the computer-assisted interview system developed for the INTERPHONE study was used for the face-to-face interviews (Cardis and Kilkenny, 1999). Subjects were asked about their mobile phone use, including the dates of starting and stopping to use each phone, the average duration and frequency of calls, and other usage patterns in chronological order. Use of the Personal Handy-phone System (PHS), a type of cordless telephone system, was also recorded. Demographic variables, medical history, and occupational history were also recorded. Clinical information on the cases was obtained from the relevant department.

When a case had over four matched controls, the latter was reduced randomly to make a 4 : 1 ratio for each case. Thus, 83 gliomas had 163 matched controls, 128 meningiomas had 229 controls, and 101 pituitary adenomas had 161 controls.

Regular mobile phone use was defined as used at least once a week for 6 months, and the reference date for phone use was set at one year before diagnosis for each case to eliminate any effect of disease in its prodromal stage; the same date being applied to matched controls. Furthermore, for regular phone users, two indices were created: cumulative length of use and cumulative call time, the former defined as the time (years) since first use, excluding any period when it was not regularly used. For recent use, either the reference date or the stop date of the last phone, whichever came first, was adopted as the end of use date. The cumulative call time was the total call durations (hours) since first use, which was the sum of call durations with all phones. Typically, the daily call duration was calculated by multiplying the average call duration per call by the number of calls per day, the call duration for each phone being the product of the daily call duration and the length of use.

An odds ratio (OR), in which the reference category was the non-user, unless otherwise specified, was calculated with the conditional logistic regression model, in which the educational level (junior high school, high school, 2-year college, 4-year college or graduate school) and marital status (married or others) were simultaneously adjusted for as categorical variables.

To examine for an association with laterality of phone use, we employed a conditional logistic regression analysis, in which EMF exposure was assumed only when the self-reported side of phone use was the same as the tumour's (ipsilateral use). A similar analysis was performed for contralateral use, with exposure assumed when the self-reported side of phone use was the opposite of the tumour's. If both ears were reported used for phone use, exposure was assumed for both analyses.

To account for the three-dimensional, spatial relationship between tumour and RF exposure distribution, we attempted to estimate the maximal SAR value inside the tumour. Since pituitary adenomas occur in the sellar region, where RF exposure is negligible, SAR was estimated only for gliomas and meningiomas as follows. Mobile phones were categorised into a small number of groups in terms of SAR distribution (Wake *et al*, 2006), since each phone model has a different intracranial SAR distribution and it is impossible to estimate the actual SAR distributions for the hundreds of mobile phones used by the cases and controls. We used the SAR distribution data from 76 phones on the Japanese market in January 2001 measured with a phantom using standard procedure for compliance testing in Japan similar to the international standard procedure (IEC 62209). Surface SAR was measured in a limited region near the phone, and cube SAR data was measured in a small three-dimensional region around the maximal SAR location. The cluster analysis was then applied to categorise the phones, in which SAR distributions were represented by location of maximal SAR, surface area larger than 50% of the maximal SAR, or depth larger than 50% of the maximal SAR. The results of categorisation depended on the conditions of phone use. Mobile phones could be classified into four categories assuming normal use condition as cheek position with antenna extracted: (1) flip/flop phones of both 800-MHz and 1.5-GHz band with an antenna in the centre; (2) flip/flop phones of 800-MHz band with an antenna on the top; (3) 1.5-GHz band phones with an antenna on top; and (4) straight phones of 800-MHz band with an antenna on top. On the basis of this finding, a hypothetical three-dimensional SAR distribution was constructed for each category of mobile phone on the above head model, Japanese numerical TARO model's head (Nagaoka *et al*, 2004). First, we estimated three-dimensional SAR distribution for each phone in TARO model’s head with the measured SAR data (Wake *et al*, 2005). The surface SAR data measured in the limited regions of the phantom was extrapolated to its whole head, and was further projected to the surface of TARO model’s head. Then, SAR in depth direction was estimated assuming exponential decay of the first three layers. Finally, SAR distribution for each phone category was obtained by averaging SAR distributions for all phones belonging to the category (Varsier *et al*, 2007).

Each mobile phone actually used was allocated to one of four categories and the hypothetical SAR distribution assigned, taking into account which ear was in contact (Varsier *et al*, 2007). The tumour location for each case was measured on a 12 computed tomography scan-cut chart model. Because of their differences, the TARO’s head was transformed to match the chart model by projection. Then the SAR value inside the tumour was estimated at a 1-cm resolution and the maximal SAR was identified for each phone. For matched controls, the maximal SAR value was estimated for the tumour location of the corresponding cases.

Three exposure indices were constructed based on the SAR distribution inside the tumour. The mean maximal SAR (mean maxSAR) was calculated for each subject by averaging the maximal SAR value over the mobile phone used by the subject. The cumulative maxSAR-year was defined as the cumulative years of use weighted by maxSAR, or calculated by summing the product of maxSAR and length of use in years for each phone over the different mobile phones used by each subject. The cumulative maxSAR-hour was defined as the cumulative call time weighted by maxSAR, or calculated by summing the product of the maxSAR and call time in hours for each phone over the different mobile phones used by each subject.

In a case-only analysis, the mean maxSAR, cumulative maxSAR-year, and cumulative maxSAR-hour were estimated for the actual and for a hypothetical tumour location, which was the mirror image of the tumour on the opposite side across the sagittal plane. The rationale was that the above SAR indices should have a higher value for the actual than for the hypothetical tumour location if RF exposure increased tumour risk. Each of the three indices was dichotomised into high and low values using the median distribution among the controls as the cut-off point: 0.0012 (glioma) and 0.0011 (meningioma) for average maxSAR; 0.0059 (glioma) and 0.0041 (meningioma) for cumulative maxSAR-year; and 0.447 (glioma) and 0.146 (meningioma) for cumulative maxSAR-hour; the McNemar test was used for comparisons.

Statistical analyses used STATA/SE version 8.2 (College Station, StataCorp, TX, USA). All statistical tests were two-sided; separate analyses were performed including and excluding the PHS system, which has a much lower emission power than mobile telephones, and as the results showed no substantial differences (data not shown), those analyses excluding the PHS system are presented here.

This study was approved by the Institutional Review Boards of the participating institutes. Its protocol was reviewed by the research group's Privacy Protection Subcommittee, which also monitored the procedures employed.

## RESULTS

The basic characteristics of the cases and controls are shown in Table 1. For time-varying items, the situations at the reference date are displayed. Cases and controls showed no substantial differences with respect to age, sex, residential area, educational level, or marital status. Educational level and marital status were considered to reflect their socioeconomic status and were selected *a priori* as confounding variables. The overall participation rate of the controls in the full study was 51.2%, but an additional 28.8% of eligible controls answered the brief survey about their mobile phone use. The age- and sex-adjusted proportions of regular users were 66.4% (glioma) and 55.1% (meningioma) in the brief survey, and were comparable with 65% (glioma) and 51.5% (meningioma) in the full study.

The adjusted OR (95% confidence interval (CI)) for regular mobile phone use was 1.22 (0.63–2.37) for glioma, 0.70 (0.42–1.16) for meningioma, and 0.90 (0.50–1.61) for pituitary adenoma, the reference date being set at 1 year before diagnosis (Table 2). The adjusted OR did not change significantly when the reference date was set at 5 years before diagnosis: 0.90 (0.47–1.72) for glioma, 1.32 (0.72–2.43) for meningioma, and 0.96 (0.52–1.79) for pituitary adenoma.

When cumulative length of use and call time were categorised into quartiles according to distribution among the controls for each tumour type, as shown in Table 2, no increasing trend was found in risks of glioma, meningioma, or pituitary adenoma. In addition, the OR for a cumulative use of 10 years or longer was 0.58 (0.09–3.86) for glioma, 1.35 (0.31–5.93) for meningioma, and 1.15 (0.22–5.18) for pituitary adenoma, although the numbers were very small: 2 cases and 6 controls for glioma; 4 cases and 4 controls for meningioma; and 4 cases and 5 controls for pituitary adenoma. The OR for a cumulative call time of 2000 h or more was 1.47 (0.41–5.28) for glioma, 0.64 (0.14–3.86) for meningioma, and 1.41 (0.46–4.37) for pituitary adenoma, again on the basis of very small numbers: 6 cases and 9 controls for glioma; 3 cases and 6 controls for meningioma; and 9 cases and 12 controls for pituitary adenoma.

No subject had used analogue-type phones alone. No difference in OR was identified for use of both analogue and digital phones *vs* digital phones only: 0.83 (0.23–3.00) *vs* 1.29 (0.66–2.53) for glioma; 1.06 (0.36–3.09) *vs* 0.67 (0.40–1.13) for meningioma; and 0.54 (0.17–1.75) *vs* 0.95 (0.53–1.71) for pituitary adenoma, respectively.

In the laterality analysis, in which it was simply assumed that exposure existed when the tumour location (left or right) matched that for phone use, the ORs for ipsilateral and contralateral use were 1.24 (0.67–2.29) and 1.08 (0.57–2.03) for glioma, and 1.14 (0.65–2.01) and 0.65 (0.37–1.13) for meningioma, respectively.

**Figure 1:**
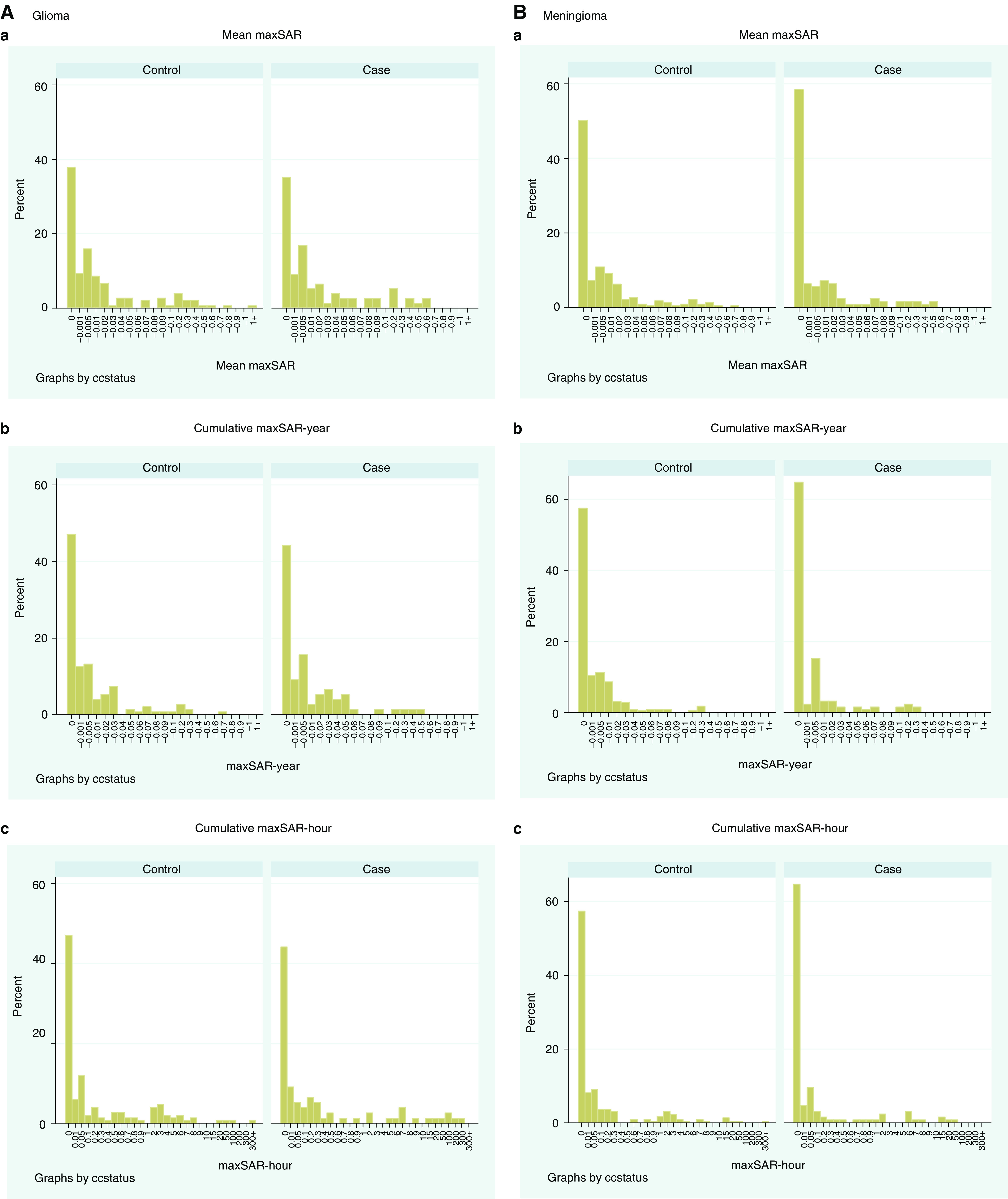
Distribution of estimated maxSAR-derived exposure indices in the tumour. (**A**) Glioma; (a) mean maxSAR; (b) cumulative maxSAR-year; (c) cumulative maxSAR-hour. (**B**) Meningioma; (a) mean maxSAR; (b) cumulative maxSAR-year; (c) cumulative maxSAR-hour.

For the analysis using maxSAR-derived exposure indices, 77 of 83 glioma cases and 125 of 128 meningioma cases with tumour location charts available, with 151 and 221 controls, respectively, were included. Mean maxSAR was estimated for each case, ranging from 1.2 × 10^−6^ to 0.0599 W kg^−1^ for glioma and 6.8 × 10^−7^ to 0.0619 W kg^−1^ for meningioma (Figure 1). The distribution of cumulative maxSAR-year and cumulative maxSAR-hour are also presented in Figure 1. The non-exposed group consisted of non-users and regular users whose mean maxSAR was extremely low (less than 1 × 10^−4^): 34 (44.2%) cases and 71 (47.0%) controls for glioma, and 81 (64.8%) cases and 127 (75.5%) controls for meningioma. The OR for exposed to non-exposed was 1.28 (0.63–2.57) for glioma and 0.72 (0.42–1.24) for meningioma. In addition, the exposed group was divided into four subgroups on the basis of the quartiles of distribution of maxSAR-derived indices among the controls. No increasing trend was found for either glioma or meningioma risk with an increase in mean maxSAR, cumulative maxSAR-year, or cumulative maxSAR-hour (Table 3).

Since the ORs for the highest quartile were all higher than unity for glioma (1.04 (0.37–2.93) for mean maxSAR, 1.75 (0.63–4.85) for cumulative maxSAR-year, and 1.55 (0.57–4.19) for cumulative maxSAR-hour), we further examined risk associated with a very high exposure. When categorised by cut-off points of 0.001 and 0.01 W kg^−1^, the adjusted OR for those with mean maxSAR over 0.01 W kg^−1^ as compared with the non-exposed was 0.87 (0.28–2.75) for glioma and 1.17 (0.40–3.39) for meningioma, indicating no substantial increase in risk. When categorised by cut-off points of 0.001, 0.01, and 0.1 W kg^−1^-year, the adjusted OR for those with cumulative maxSAR-year over 0.1 W kg^−1^-year as compared with the non-exposed was 0.63 (0.14–2.93) for glioma and 2.72 (0.47–15.98) for meningioma, again indicating no substantial increase in risk. When categorised by cut-off points of 0.1, 1, and 10 W kg^−1^-hour, on the other hand, the adjusted OR for the highest *vs* lowest category increased, although not significantly, to 5.84 (0.96−35.60) for glioma, on the basis of 7 cases (9.1%) and 4 controls (2.7%) in the highest category. No increase in risk was found for meningioma: 1.14 (0.28–4.61), on the basis of 4 cases (3.2%) and 6 controls (2.7%).

In the case-only analysis, no differences were identified when three dichotomised maxSAR-derived indices were compared between the actual and hypothetical opposite tumour location (as shown in Table 4).

## DISCUSSION

No consistent increase was observed in the overall risk of glioma or meningioma among mobile phone users, nor increasing trend in risk in relation to cumulative length of use or cumulative call time. We further estimated the SAR in the intracranial space with a resolution of 1 cm^3^, and constructed three SAR-derived exposure indices (mean maxSAR, cumulative maxSAR-year, and cumulative maxSAR-hour) to estimate the cumulative exposure level inside the tumour as precisely as possible. Again, no substantial increase in risk was observed for glioma or meningioma. Maximal SAR inside the tumour was estimated to be lower than 0.1 W kg^−1^ in all eligible cases, far below the ICNIRP’s recommended value of 2 W kg^−1^ for localised SAR (head and trunk) in the general population.

This is to our knowledge, the first epidemiological study to take into account the different exposure levels inside the intracranial space. Exposure is localised close to the relevant ear, whereas glioma and meningioma develop at a variety of sites. Estimation of the actual exposure level inside the tumour is essential, therefore, to avoid exposure misclassification, which bias risk estimates towards the null. Nevertheless, recall bias is a possibility and awareness of tumour location among the cases might have affected their recall concerning which ear they used for mobile phone calls. The non-significant increase in OR of 5.84 for those with cumulative maxSAR-hour over 10 W kg^−1^-hour as compared with the non-exposed group could reflect recall bias, since increased OR was observed only for this heavily exposed group; it needs to be interpreted with caution.

We masked the study’s purpose during recruitment, since this could have affected participation differentially among cases and controls, with consequently a biased risk estimate. During the random digit dialling control selection, we tried to ensure that this did not vary between those who were regularly at home and those who were out more often: mobile phone users tend to be more active and busier, staying at home for shorter periods than non-users, and risk could be overestimated if controls included an unbalanced number of non-users. Although participation among controls in the full interview remained around 50%, we were able to examine its impact on risk estimates by using the information obtained from the brief questionnaire completed by non-participating controls. The age- and sex-adjusted proportions of regular mobile phone users among the control candidates who did not participate in the full interview but agreed to respond to the brief questionnaire (28.8% of eligible controls) were 66.4% (glioma) and 55.1% (meningioma) as compared with 65.0% (glioma) and 51.5% (meningioma) for the controls who participated in the full, face-to-face interview (51.2% of eligible controls), respectively, indicating that the interviewed controls did not constitute a biased sample in terms of mobile phone use. Adjusted ORs for regular mobile phone users did not vary significantly, irrespective of whether the controls were taken as those who responded to the brief questionnaire or those who took part in the full interview (the ORs were 1.21 for glioma and 0.70 for meningioma).

To date, several case–control studies have been reported from the United States (Muscat *et al*, 2000; Inskip *et al*, 2001), Finland (Auvinen *et al*, 2002), and Sweden (Hardell *et al*, 1999, 2002, 2003, 2004) before the INTERPHONE study. Two US studies showed negative results for the overall risk of brain tumours, whereas in a Finnish population register-based study, the OR for glioma with relation to ever use of analogue mobile phones was 2.1 (95% CI: 1.3–3.4). Risk was estimated for the left/right laterality of the tumour or for the affected lobe in relation to that of mobile phone use, but no increased risk was observed for tumours on the same side as phone use. In a series of Swedish studies, the risk of tumours in the temporal area on the same side as that used for mobile phone calls was increased for analogue phones, OR 2.3 (95% CI: 1.2–4.1). However, no increased risks by histological types were observed (Hardell *et al*, 2003).

Four studies from the INTERPHONE study showed no increased overall risk of glioma or meningioma in relation to regular mobile phone use (Christensen *et al*, 2004, 2005; Hepworth *et al*, 2006; Schuz *et al*, 2006), although glioma risk increased non-significantly (OR=2.20; 95% CI: 0.94–5.11) among long-term users (10 years or more), but no excess risk was found for temporal lobe tumours, considered to be exposed to the highest radio frequency (RF)–EMF (Schuz *et al*, 2006). In a Swedish study, the OR for glioma on the same side as mobile phone use increased to 1.8 (95% CI: 0.8–3.9) among long-term (⩾10 years) mobile phone users, but the corresponding OR for glioma on the opposite side was found to decrease to 0.6 (95% CI: 0.3–1.4) among long-term users (Lönn *et al*, 2005). In a UK study, the OR for glioma on the same side as mobile phone use was 1.24 (95% CI: 1.02–1.52), but for gliomas on the opposite side, it decreased significantly to 0.75 (95% CI: 0.61–0.93) (Hepworth *et al*, 2006). In both studies, it was suggested that such ‘complementary risks’ above and below unity could reflect recall bias, in which glioma patients tended to recall that they used mobile phones on the same side as the tumour location simply because they knew where the tumour was. In summary, the findings to date on mobile phone use and glioma and meningioma risks are inconsistent.

Most studies of the non-thermal effects of RF–EMF indicate no direct DNA effects such as mutagenicity or genotoxicity of RF–EMF exposure in the range of 800–1900 MHz and an SAR of less than 2 W kg^−1^. There is also little evidence for indirect DNA effects, including alterations in gene expression, cell proliferation, or apoptosis (Laszlo *et al*, 2005; Chauhan *et al*, 2006; Lixia *et al*, 2006; Qutob *et al*, 2006; Joubert *et al*, 2007). Thus, laboratory studies do not support the possibility that mobile phone use increases the risk of brain tumours.

In conclusion, we observed no increase in overall risk of glioma or meningioma in relation to regular mobile phone use among our Japanese subjects.

## Figures and Tables

**Table 1:** Case–control comparison of basic characteristics at reference date

	**Glioma**		**Meningioma**		**Pituitary adenoma**	
	**Case no.**	**Control no.**	**Case no.**	**Control no.**	**Case no.**	**Control no.**
*Age (years)*						
30–39	27	56	14	26	26	43
40–49	18	38	32	57	19	30
50–59	27	48	54	97	49	73
60–69	11	21	28	49	7	15
						
*Sex*						
Male	44	85	29	48	62	101
Female	39	78	99	181	39	60
						
*Education*						
Junior high	10	10	7	20	6	9
High	40	78	91	150	56	74
College+	33	75	30	59	39	78
						
*Marital status*						
Married	59	125	95	174	71	134
Others	24	38	33	55	30	27
						
*Residential area*						
Tokyo (metro)	36	63	61	96	49	71
Tokyo (municipal)	9	22	14	27	13	24
Chiba	8	20	12	20	9	12
Kanagawa	11	25	24	41	13	20
Saitama	19	33	17	45	17	34
						
*Smoking status*						
Non-smoker	59	125	95	174	71	134
Ex-smoker	24	38	33	55	30	27
Current smoker	24	38	33	55	30	27
						
*Timing of interview*						
December 2000–November 2001	25	8	32	20	32	16
December 2001–November 2002	17	31	27	45	22	40
December 2002–November 2003	29	76	36	59	32	40
December 2003–November 2004	12	39	33	95	15	59
December 2004–March 2005	0	9	0	10	0	6

**Table 2:** Case–control comparison of the indices of mobile phone use

	**Glioma**			**Menigioma**			**Pituitary adenoma**		
	**Case no.**	**Control no.**	**OR (95% CI)**	**Case no.**	**Control no.**	**OR (95% CI)**	**Case no.**	**Control no.**	**OR (95% CI)**
*Mobile phone use*									
Non-user	27	57	1.0	73	111	1.0	39	56	1.0
Regular user	56	106	1.22 (0.63–2.37)	55	118	0.70 (0.42–1.16)	62	105	0.90 (0.50–1.61)
									
*Cumulative length of use in years* a									
Non-user	27	57	1.0	73	111	1.0	39	56	1.0
Lowest	11	25	0.92 (0.37–2.28)	6	28	0.39 (0.10–1.01)	14	25	0.86 (0.39–1.88)
Mid-low	21	27	1.65 (0.70–3.90)	13	27	0.70 (0.31–1.58)	13	27	0.75 (0.31–1.81)
Mid-high	17	25	1.85 (0.78–4.40)	16	33	0.73 (0.35–1.53)	22	26	1.64 (0.74–3.66)
Highest	7	29	0.60 (0.20–1.78)	20	30	1.05 (0.52–2.11)	13	27	0.75 (0.31–1.82)
	*P* for trend=0.743			*P* for trend=0.800			*P* for trend=0.885		
									
*Cumulative call time in hours* b									
Non-user	27	57	1.0	73	111	1.0	39	56	1.0
Lowest	15	26	1.57 (0.66–3.74)	12	28	0.74 (0.33–1.67)	15	26	1.00 (0.46–2.16)
Mid-low	14	27	0.88 (0.35–2.22)	15	31	0.66 (0.32–1.37)	14	26	0.97 (0.40–2.32)
Mid-high	9	25	0.90 (0.34–2.36)	11	29	0.55 (0.24–1.26)	12	26	0.72 (0.31–1.70)
Highest	18	28	1.74 (0.71–4.26)	17	30	0.92 (0.43–1.96)	21	27	1.33 (0.58–3.09)
	*P* for trend=0.483			*P* for trend=0.356			*P* for trend=0.865		
									
*Type of mobile phone used*									
Non-user	27	57	1.0	73	111	1.0	39	56	1.0
Analogue+digital	6	13	0.83 (0.23–3.00)	7	9	1.06 (0.36–3.09)	5	14	0.54 (0.17–1.75)
Digital	50	93	1.29 (0.66–2.53)	48	109	0.67 (0.40–1.13)	57	91	0.95 (0.53–1.71)
									
*Tumour laterality and side of mobile phone use*									
Reference	49	113	1.0	97	178	1.0	(*not done)*		
Ipsilateral use^c^	31	50	1.24 (0.67–2.29)	31	50	1.14 (0.65–2.01)			
Reference	55	114	1.0	102	162	1.0	(*not done)*		
Contralateral use^d^	25	49	1.08 (0.57–2.03)	26	60	0.65 (0.37–1.13)			

Abbreviations: CI=confidence interval; OR=odds ratio adjusted for education and marital status.

a Cut-off points for quartiles (divided on the basis of the distribution of the control group): 2.2, 4.7, and 6.5 years for glioma; 1.6, 3.2, and 5.2 years for meningioma; 2.4, 4.5, and 7.2 years for pituitary adenoma.

b Cut-off points for quartiles (divided on the basis of the distribution of the control group): 32, 160, and 620 h for glioma; 19, 61, and 260 h for meningioma; 39, 190, and 560 h for pituitary adenoma.

c Ipsilateral use: tumour location (left/right) was the same as the side of mobile phone use.

d Contralateral use: tumour location (left/right) was the opposite as the side of mobile phone use.

**Table 3:** Risk of brain tumour with relation to mobile phone use, considering estimated maximal SAR in the tumour as an exposure index

	**Glioma**			**Meningioma**		
	**Case no.**	**Control no.**	**OR (95% CI)**	**Case no.**	**Control no.**	**OR (95% CI)**
*Mean maxSAR* a						
Non-exposed^b^	34	71	1.0	81	127	1.0
Lowest	10	20	0.91 (0.32–2.56)	7	23	0.40 (0.15–1.09)
Mid-low	7	20	0.81 (0.26–2.53)	9	24	0.48 (0.17–1.32)
Mid-high	15	20	2.98 (0.98–9.01)	13	23	0.94 (0.39–2.29)
Highest	11	20	1.04 (0.37–2.93)	15	24	1.10 (0.50–2.41)
	*P* for trend=0.402			*P* for trend=0.402		
Non-exposed^b^	34	71	1.0	81	127	1.0
<0.001	17	37	0.94 (0.40–2.24)	16	44	0.46 (0.21–1.00)
0.001–0.01	17	27	2.30 (0.86–6.19)	21	38	0.86 (0.41–1.80)
⩾0.01	9	16	0.87 (0.28–2.75)	7	12	1.17 (0.40–3.39)
	*P* for trend=0.492			*P* for trend=0.749		
						
*Cumulative maxSAR-year* c						
Non-exposed^b^	34	71	1.0	81	127	1.0
Lowest	10	20	1.08 (0.37–3.16)	3	23	0.18 (0.05–0.63)
Mid-low	10	20	1.26 (0.46–3.43)	18	24	1.10 (0.48–2.50)
Mid-high	8	20	1.07 (0.33–3.45)	8	23	0.56 (0.21–1.48)
Highest	15	20	1.75 (0.63–4.85)	15	24	1.07 (0.48–2.36)
	*P* for trend=0.306			*P* for trend=0.904		
Non-exposed^b^	34	71	1.0	81	127	1.0
<0.001	7	19	0.66 (0.21–2.09)	3	23	0.17 (0.05–0.61)
0.001–0.01	14	26	1.53 (0.61–3.85)	23	44	0.76 (0.37–1.54)
0.01–0.1	18	28	2.09 (0.75–5.83)	13	22	0.93 (0.39–2.20)
⩾0.1	4	7	0.63 (0.14–2.93)	5	5	2.72 (0.47–15.98)
	*P* for trend=0.332			*P* for trend=0.904		
						
*Cumulative maxSAR-hour* d						
Non-exposed^b^	34	71	1.0	81	127	1.0
Lowest	8	20	0.89 (0.30–2.64)	9	23	0.63 (0.26–1.52)
Mid-low	16	20	1.82 (0.73–4.49)	14	24	0.78 (0.33–1.84)
Mid-high	6	20	0.71 (0.23–2.18)	10	23	0.76 (0.33–1.78)
Highest	13	20	1.55 (0.57–4.19)	11	24	0.70 (0.30–1.63)
	*P* for trend=0.437			*P* for trend=0.402		
Non-exposed^b^	34	71	1.0	81	127	1.0
<0.1	14	30	1.09 (0.44–2.70)	22	46	0.67 (0.34–1.32)
0.1–1	14	22	1.30 (0.52–3.23)	9	24	0.66 (0.28–1.59)
1–10	8	24	0.92 (0.31–2.69)	9	18	0.71 (0.27–1.89)
⩾10	7	4	5.84 (0.96–35.60)	4	6	1.14 (0.28–4.61)
	*P* for trend=0.244			*P* for trend=0.484		

Abbreviations: CI=confidence interval; OR=odds ratio adjusted for education and marital status; SAR=specific absorption rate.

a Cut-offs for quartiles (on the basis of the distribution of the control group): 0.00036, 0.0012, and 0.008 for glioma; 0.00049, 0.0011, and 0.0048 for meningioma.

b Non-exposed group includes mobile phone users whose maximal SAR was estimated to be <0.0001 W kg^−1^.

c Cut-offs for quartiles (on the basis of the distribution of the control group): 0.0012, 0.0059, and 0.025 for glioma; 0.001, 0.0041, and 0.014 for meningioma.

d Cut-offs for quartiles (on the basis of the distribution of the control group): 0.028, 0.447, and 2.18 for glioma; 0.014, 0.146, and 1.12 for meningioma.

**Table 4:** Case-only analysis to compare the distribution of maximal SAR-related indices within the tumour and its axis-symmetrical location^a^

	**Glioma**			**Meningioma**	
	**Axis-symmetrical tumour location^b^**			**Axis-symmetrical tumour location^b^**	
*Mean maxSAR*					
	⩾0.0012	<0.0012		⩾0.0011	<0.0011
Actual tumour ⩾0.0012	11	15	Actual tumour ⩾0.0011	17	11
Location <0.0012	11	13	Location <0.0011	10	14
		*P*=0.433			*P*=0.827
					
*Cumulative maxSAR-year*					
	⩾0.0059	<0.0059		⩾0.0041	<0.0041
Actual tumour ⩾0.0059	11	12	Actual tumour ⩾0.0041	12	11
Location <0.0059	10	17	Location <0.0041	12	17
		*P*=0.670			*P*=0.835
					
*Cumulative maxSAR-hour*					
	⩾0.447	<0.447		⩾0.146	<0.146
Actual tumour ⩾0.447	10	9	Actual tumour ⩾0.146	9	12
Location <0.447	9	22	Location <0.146	9	22
		*P*=1.000			*P*=0.664

Abbreviation: SAR=specific absorption rate.

a The study subjects analysed here were limited to the cases with regular mobile phone use whose maximal SAR distribution was estimated.

b Hypothetical tumour location on the opposite side of the actual tumour across the sagittal plane.
